# MRI Guided Brain Stimulation without the Use of a Neuronavigation System

**DOI:** 10.1155/2015/647510

**Published:** 2015-08-27

**Authors:** Ehsan Vaghefi, Peng Cai, Fang Fang, Winston D. Byblow, Cathy M. Stinear, Benjamin Thompson

**Affiliations:** ^1^Department of Optometry and Vision Science, University of Auckland, Building 502, Level 4, 85 Park Road, Grafton, Auckland 1023, New Zealand; ^2^Department of Psychology, Peking University, Haidian Road, Haidian, Beijing 100871, China; ^3^Department of Sport and Exercise Science, University of Auckland, Symonds Street, Auckland 1023, New Zealand; ^4^Department of Medicine, University of Auckland, Symonds Street, Auckland 1023, New Zealand; ^5^School of Optometry and Vision Science, University of Waterloo, 200 Columbia Street W, Waterloo, ON, Canada N2L 3G1

## Abstract

A key issue in the field of noninvasive brain stimulation (NIBS) is the accurate localization of scalp positions that correspond to targeted cortical areas. The current gold standard is to combine structural and functional brain imaging with a commercially available “neuronavigation” system. However, neuronavigation systems are not commonplace outside of specialized research environments. Here we describe a technique that allows for the use of participant-specific functional and structural MRI data to guide NIBS without a neuronavigation system. Surface mesh representations of the head were generated using Brain Voyager and vectors linking key anatomical landmarks were drawn on the mesh. Our technique was then used to calculate the precise distances on the scalp corresponding to these vectors. These calculations were verified using actual measurements of the head and the technique was used to identify a scalp position corresponding to a brain area localized using functional MRI.

## 1. Introduction

Noninvasive brain stimulation (NIBS) techniques such as repetitive transcranial magnetic stimulation (rTMS) and transcranial direct current stimulation (tDCS) allow for the temporary modulation of neural activity within the human brain. rTMS involves the induction of weak electrical currents within targeted regions of the cortex via brief, time-varying magnetic fields produced with a hand-held coil [1]. tDCS employs head-mounted electrodes, which allow for a weak direct current to interact with the underlying cortex [2]. NIBS can be used to investigate the role of individual brain areas in specific cognitive, behavioral, or perceptual processes [1]. In addition, these techniques are being investigated from a clinical perspective and current evidence suggests that NIBS may be applicable to the treatment of multiple neurological and psychiatric disorders [3, 4].

Studies involving the use of NIBS begin by selecting a target brain area for stimulation. This process is typically informed by evidence from brain imaging, animal neurophysiology, or studies involving neurological patients. Subsequent steps include the selection of appropriate stimulation parameters and ensuring that the stimulation is delivered to the correct brain area. This latter point is particularly important as the stimulation effects are most pronounced in close proximity to the rTMS coil and tDCS electrodes [5]. Therefore, accurate, participant-specific localization of stimulation sites on the scalp is required for optimal stimulation [6].

A number of approaches can be used to identify the correct scalp position for stimulation. Single pulse TMS can be used to activate specific regions of the primary motor cortex resulting in motor evoked potentials (MEPs) within the corresponding peripheral muscle [7]. The scalp location that evokes the strongest MEP can then be used as the location for rTMS or tDCS. A comparable technique also exists for the visual cortex whereby single pulse TMS of the occipital pole can be used to evoke the percept of a phosphene [8]. The scalp location that induces the most robust phosphene or a phosphene in a specific visual field location can be used for visual cortex stimulation. A similar technique can be used for motion sensitive, extra-striate visual area V5 whereby TMS can be used to induce moving phosphenes [9]. It has been shown that this technique is in good agreement with localization of V5 using functional magnetic resonance imaging [10]. However, it is not possible to use this approach outside of the motor and visual cortices because most brain regions do not produce acute neurophysiological or perceptual effects in response to single pulse TMS.

An alternative technique for identifying participant-specific stimulation sites on the scalp is the 10–20-electrode system, which was originally designed for positioning EEG electrodes [11]. This approach defines a grid of positions on the scalp that are separated by 10% or 20% of the distance between anatomical landmarks such as the nasion and the inion. This approach has been used successfully in a large number of brain stimulation studies; however, the mapping of particular 10–20 system locations to specific brain areas can vary across participants [12].

Another alternative is to use structural and functional brain imaging techniques to localize specific brain areas in individuals with millimetre resolution. A number of frameless stereotactic navigation systems exist for real-time coregistration of a participant to their own MRI images. Tools such as a “pointer” or a TMS coil can also be registered within the volume. These systems typically involve ultrasound devices or infrared cameras and a number of reference targets mounted on the head and NIBS apparatus. When used in combination with structural and functional MRI images these “neuronavigation” systems allow for precise identification of the scalp position corresponding to a particular brain area [13].

The combination of brain imaging and a neuronavigation system is the current gold standard in the field of NIBS [14] and may improve the results of NIBS-based therapeutic interventions [15–20]; however, there are some disadvantages. These include difficulty in using these systems for studies of posterior brain areas that can fall outside of the neuronavigation system's field of view and, most importantly, the high cost of these systems, which can exceed $50,000. Techniques have been described that allow NIBS to be targeted using generic MRI datasets [21] or when structural but not functional MRI data are available for individual participants [22]. Furthermore, techniques for identifying optimal scalp locations for stimulation based on individual participant's neuroanatomy are also available [23]. However, each of these approaches requires the use of a neuronavigation system. Here we describe a technique that allows the use of individual structural and functional MRI to guide NIBS in the absence of a neuronavigation system. The approach is based on vectors drawn on a mesh that is morphed to participant-specific MRI data. These mesh vectors are then transposed to the participant's head by converting them to head measurements anchored to anatomical landmarks. We report comparisons between measurements made using our technique and actual head measurements. We also give an example of how the technique can be used in combination with fMRI to localize a stimulation site for visual area V5 in a single subject. Visual area V5 was chosen for this example as it can be readily localized using fMRI and the corresponding scalp position cannot be identified based on a single anatomical landmark. Therefore, a number of measurements are required to triangulate the correct scalp location for stimulation. A MATLAB package is also provided, which allows the use of our technique in conjunction with the commercially available Brain Voyager software package or any other software platform that supports the morphing of meshes to MRI data.

## 2. Methods

### 2.1. Participants

Six healthy adult participants (5 male and 1 female, mean age 32 years) provided written informed consent and took part in this study. fMRI data were collected from one participant to provide a participant-specific example of how our technique can be used in combination with a functional localizer. All study procedures were approved by the institutional ethics review board and were in accordance with the Declaration of Helsinki.

### 2.2. Magnetic Resonance Imaging

MRI data were acquired using a 3.0 Tesla Philips Achieva scanner equipped with an 8-channel head coil. A T1-weighted 3D turbo field-echo anatomical volume (1000 ms inverted prepulse, 1 × 1 × 1 mm^3^ voxel resolution, 180 sagittal slices, 2.7 ms TE, 5.9 ms TR, and 8° flip angle) was acquired for each participant. For functional localization of V5, four functional scans were conducted using a T2^*^-weighted gradient echo, EPI sequence (TR = 2 s, TE = 30 ms, and flip angle = 65°) to acquire 160 volumes constructed from 39 axial slices covering the whole brain at voxel resolution of 3 × 3 × 3 mm. During the functional scans the participant viewed static and dynamic radial grating stimuli (10° × 20°, 0.4 cycles per degree, 3% contrast, and 9 Hz temporal frequency when moving) presented on a mean luminance background (5 cd/m^2^) [24]. Dynamic gratings were presented in 20-second blocks separated by 20-second blocks of stationary gratings. There were 6 dynamic blocks per scan. During each dynamic block the participant fixated centrally and performed a task whereby they judged the relative speed of two grating movements, one centripetal and one centrifugal. Each movement lasted 250 ms with an interstimulus interval of 50 ms and a behavioural response time of 1450 ms. These stimuli resulted in robust activation in area V5.

**Figure 1:**
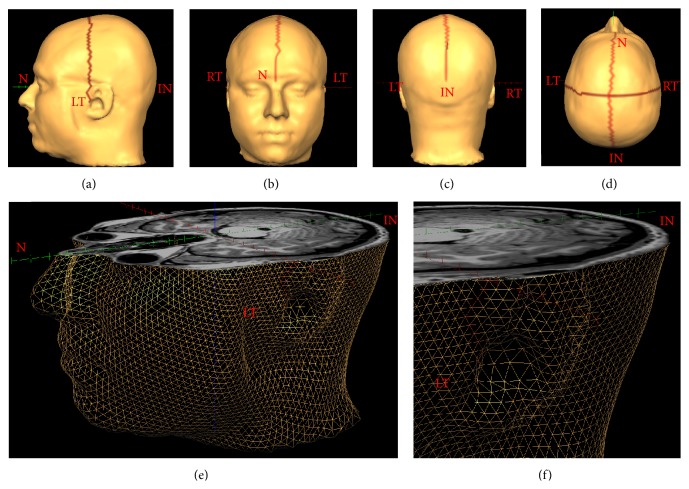
A 3D mesh morphed to the structural MRI data of a representative participant. Panels (a)–(d) show the anatomical landmarks that were used as anchor points for scalp distance calculations marked on a T1-volume surface mesh created using Brain Voyager. N: nasion, RT and LT: right and left tragi, respectively, and IN: inion. The lines connecting the anatomical landmarks are “patches of interest” (POIs) drawn in Brain Voyager that link adjacent triangles in the mesh. Panels (e) and (f) show close-up views of the mesh without the surface coloring. The mesh has been cut axially at the level of the inion. The smooth surface of the head is represented using triangular elements and each of these elements is defined by its tricorners.

MRI data were analyzed using the commercial Brain Voyager QX package (http://www.brainvoyager.com/). A 3D mesh of the head was generated using the mesh functions within Brain Voyager applied to the native space T1 anatomical volume after AC-PC alignment (Figure 1). Functional data were corrected for head movement, high pass filtered, and aligned to the AC-PC aligned anatomical images using subroutines within Brain Voyager. A general linear analysis was conducted and the results were visualized as t-maps on the anatomical image. Area V5 was identified as a region in the appropriate anatomical location that responded significantly more strongly to dynamic than static grating stimuli (FDR corrected *q* < 0.01). The precise location of V5 was defined as the location of the peak voxel within the V5 region.

### 2.3. Comparison of Measurements Made on the Surface Mesh and the Head

Four anatomical landmarks were identified on each surface mesh: the nasion, the left and right tragi, and the inion (Figures 1(a)–1(d)). The shortest paths between the nasion and inion and the left and right tragi that passed through the center point of the head (Cz) were then marked on the surface of the 3D mesh and exported as “patches of interest” (POIs) within Brain Voyager. After this, the *x*, *y*, and *z* coordinates of the mesh nodes that formed the POI were exported from Brain Voyager in XLS format and read into the MATLAB analysis environment for distance calculations. The actual distances between the two tragi and the nasion and inion were also measured for each participant using a tape measure. An investigator masked to the results of the MATLAB analysis made these measurements.

### 2.4. MATLAB Operations

A Graphical User Interface was created in MATLAB to import the coordinate matrix of the POI exported from the Brain Voyager environment. Since the aim was to develop a widely applicable tool, the software does not require the use of Brian Voyager. Rather, the software is capable of reading a coordinate matrix from a text/MS-Excel file as this format is an export option in most image postprocessing software packages. The file must have three columns (*x*, *y*, *z*), which conform to the following format:

(1) $$\begin{array}{c} \text{Noda}{\text{l}}_{\text{vector}}=\left({\text{Node}\left(n\right)}_{x},{\text{Node}\left(n\right)}_{y},{\text{Node}\left(n\right)}_{z}\right). \end{array}$$

Here, *n* is the index of the nodal coordinate in the vector matrix. Subscripts *x*, *y*, and *z* indicate the Cartesian tricoordinates of the vector's nodal points.

**Figure 2:**
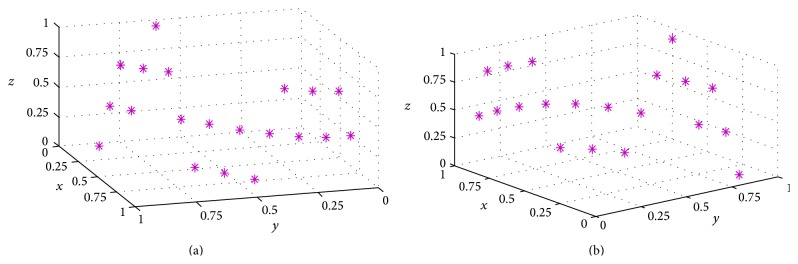
Graphical representations of the nodes making up a path along the surface of a head mesh created in Brain Voyager. (a) and (b) are two different views of the same 3D path. The axis values are 3D vector coordinates normalized to the length of the whole vector.

The MATLAB code opens the text-based input file and searches for the first line of the nodal coordinate series. Next, it reads consecutive coordinates until the pattern is broken; that is, no further coordinates are listed. The nodes of the POI/vector can then be viewed immediately in 3D (Figure 2).

There are two main issues to be addressed when calculating scalp distances from POIs measured on a surface mesh. (1) The majority of packages that provide surface meshes (Brain Voyager included) export POIs across the mesh in a proprietary format that makes it difficult to identify adjacent points along the path. (2) The use of tetrahedral elements in the mesh produces “jagged” POIs that do not exactly represent the smooth surface of the head. These two issues were resolved using the following steps.

**Figure 3:**
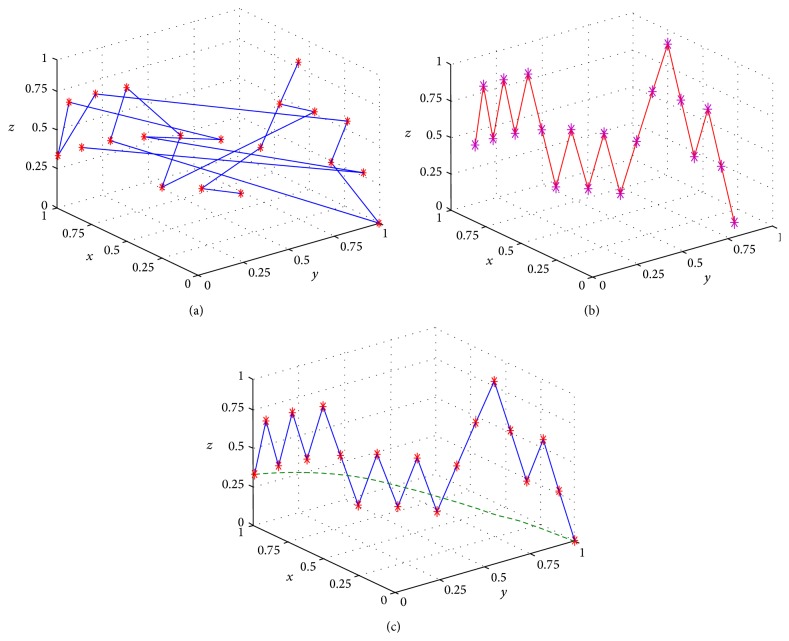
Processing of a path retrieved from a POI drawn on a surface mesh generated by Brain Voyager. (a) The randomized order of vertices provided by the Brain Voyager output. (b) The same vertices after reordering. The jagged path resulting from the triangulation of the mesh is apparent. (c) A curve fitted to the vertices (in dashed green) allows the length of the path to be accurately calculated. The axis values are normalized POI vector coordinates in 3D.

Having imported the coordinate matrix of the POI vector (Figure 2), the code first identifies the end points of the vector (i.e., nodal positions at the two ends of the vector). This is achieved by brute force comparison of pairs of nodal coordinates (i.e., marching along the POI vector), in order to find the outmost couple. A smoothed polynomial is then fitted to the two endpoints in 3 dimensions, which provides a reference for identifying adjacent nodal points in the vector. The nodal coordinate list is then sorted along the estimated trajectory of the POI vector (Figure 3). Finally, a second smoothed polynomial is fitted to the sorted nodal coordinate list in three dimensions, which removes the jaggedness of the path (Figure 3(c)). The length of the second fitted polynomial provides an estimate of the length of the vector on the participant's head. After POI vector length calculation, the imported coordinate matrix and the measured pathway are plotted in the GUI.

**Figure 4:**
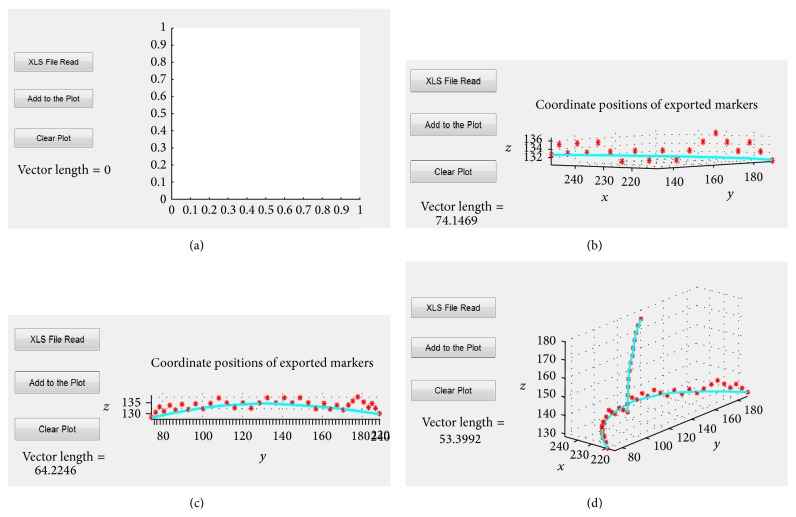
The Graphical User Interface that supports the import and plotting of POIs drawn on a surface mesh. Panel (a) shows the starting view and each consecutive panel ((b)–(d)) shows the addition of a new 3D path to the GUI. This is achieved by reading in XLS files containing the path data. The length of the most recently loaded 3D path is shown in the lower left corner of the GUI in mm.

The GUI allows multiple POIs to be imported from a single mesh and viewed simultaneously (Figure 4). The GUI also allows 3D viewing of the POI and supports 3D zooming and rotation (Figure 4).

## 3. Results

The measurements made using MATLAB were in good agreement with those made manually using a tape measure (Table 1). There were no statistically significant differences between the two sets of measurements (nasion-inion *t*(5) = 1, *P* = 0.4; tragus-tragus *t*(5) = 0.8, *P* = 0.5) and intraclass correlation (ICC) indicated that the two sets of measurements were closely related (nasion-inion ICC = 0.97, *P* < 0.001; tragus-tragus ICC = 0.98, *P* < 0.001).

Overall, our MATLAB application appeared to be capable of accurately measuring the length of the selected and exported POIs.

### 3.1. Combination of the Technique with fMRI Data

**Figure 5:**
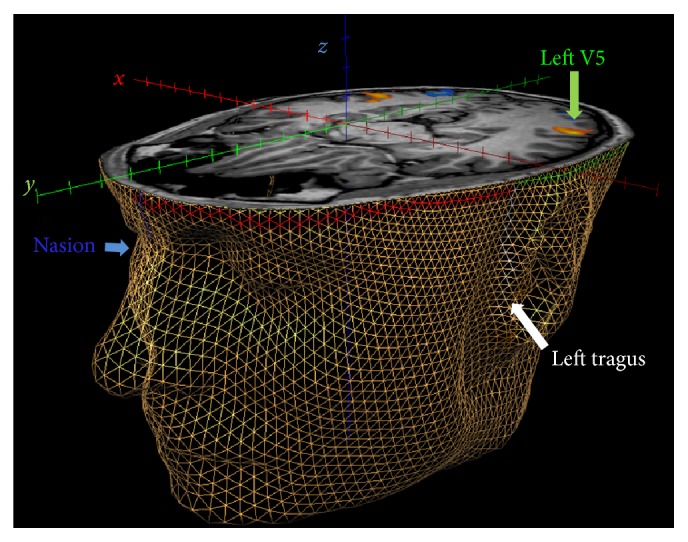
Localization of a scalp position above V5 in the left hemisphere. The axial cut through the Brain Voyager mesh was positioned to reveal the most active voxel in left V5. The lines drawn on the mesh show the POIs that were used to identify the scalp location corresponding to left V5. Blue: nasion to transverse plane, white: tragus to transverse plane, red: intersection of nasion vector and transverse plane to intersection of tragus vector and transverse plane, and green: extension of the vector to the scalp position above area V5. Orange regions indicate areas of functional activation in response to the V5 localization scans. See the main text for a detailed description of this procedure.

To assess whether the technique could be used in combination with fMRI localization data we generated scalp measurements to localize the scalp position directly above V5 within the left hemisphere of one participant (Figure 5). The procedure was as follows.

1. The functional MRI data were coregistered to the anatomical MRI data and analyzed to identify V5 (see Methods section). The 3D statistical map was then visualized within the anatomical volume and a mesh was morphed to the surface of the anatomical volume using the mesh tools within Brain Voyager.
2. The mesh was cut along the transverse plane that contained the peak V5 voxel for the left hemisphere (as shown in Figure 5).
3. A POI was drawn from the nasion to the top of the cut mesh (blue POI in Figure 5).
4. A second POI was from the left tragus to the top of the cut mesh (white POI in Figure 5).
5. A third POI was drawn to connect the uppermost points of the nasion and tragus POIs (red POI in Figure 5). This POI was then extended to the scalp position that was directly above the most active voxel in the V5 (green POI in Figure 5).
6. The MATLAB toolbox was used to calculate the length of each POI on the participant's head.
7. A hairnet was placed on the participant and tape was used to secure the net so that it was stretched tightly across the head. A line that was of the same length as the POI anchored to the nasion (blue in Figure 5) was then drawn vertically upwards from the participant's nasion using a tape measure and marker pen. The POI anchored to the left tragus (white in Figure 5) was transposed to the participant's head in the same way.
8. A line was drawn connecting the top points of the nasion and tragus POIs. The length of this line was compared to the length of the corresponding mesh POI (red in Figure 5) to ensure that the length calculations were accurate. In agreement with the data shown in Table 1, the distance between the two points on the mesh was identical to the manually measured distance on the participant's head.
9. The line connecting the nasion and tragus POIs was extended by the length of the corresponding mesh POI (green in Figure 5) to identify the scalp position above V5.

## 4. Discussion

NIBS techniques such as rTMS and tDCS are becoming widely used in both basic science and clinical research. Both of these techniques are considered noninvasive because rTMS utilizes magnetic induction and tDCS uses nonpenetrating surface electrodes to induce electrical currents within superficial areas of the cortex. This means that direct access to the brain is not required as the stimulation can be delivered from the scalp. However, it also means that identifying the participant-specific scalp location that corresponds to the target brain area can be challenging. It is well established that structural and functional MRI can be used to assist in identifying the correct location for stimulation [25–28] although a specialized “neuronavigation” system is typically required to coregister the participant to their MRI data. The aim of this study was to develop a technique that would allow for MRI guided NIBS without the need for a commercially available neuronavigation system. We found that it was possible to accurately estimate scalp distances using POIs drawn on a surface mesh derived from participant-specific T1 volumes. When anchored to anatomical landmarks, these distances could be used to locate the scalp position corresponding to a specific region of neural activity identified using functional MRI.

The technique we describe here was not designed to be a replacement for neuronavigation systems that have a number of benefits. These include real-time assessment of TMS coil position, estimates of induced current flow, and the elimination of manual measurement error (although manual registration of the head and MRI data is still required). However, neuronavigation systems are not commonplace outside of specialist research environments and therefore alternative ways of utilizing MRI data to optimize NIBS are desirable.

Previous studies have calculated geodesic distances between scalp landmarks using surface mesh representations of the head in order to guide NIBS [29–32]. Our proposed technique uses a comparable approach but unlike those used in the above studies, our tool is platform-independent and open source. Specifically, although we have implemented our technique using Brain Voyager, the principles we describe could be applied to data from any software that provides mesh vectors. It is also possible that this technique will be of use for other applications where scalp positions corresponding to specific brain areas are required. Examples include the combination of fMRI and EEG data and accurate positioning of near infrared spectroscopy apparatus. An important next step in the development of this approach will be to compare the scalp locations that are generated by our technique with those identified using commercial neuronavigation systems in a large group of participants. A comparison of MEP and phosphene induction between the two techniques will also be important.

The main advantages of our technique are its low cost and platform-independence (i.e., it can be used with any software that allows mesh morphing to MRI data). However, there are a number of limitations. For example, neuronavigation systems allow easy targeting of the same stimulation site across multiple sessions. Our technique requires remeasurement of the head for each session and this process may be prone to error. In addition, our technique allows for a stimulation site to be transposed from MRI data to the participant's head; however the selection of the optimal stimulation site itself is not supported. This issue is also relevant to the use of neuronavigation systems. Selection of the optimal stimulation site is a complex process as the electrical current generated by NIBS techniques interacts with the head and brain anatomy in ways that are unique to each participant [33–35]. A number of techniques for identifying optimal NIBS sites based on MRI data have been developed. These could be combined with our approach for transposing stimulation sites to the head to further improve the targeting of NIBS when neuronavigation systems are not available.

## Figures and Tables

**Table 1:** Comparison of the manual head measurements and the MATLAB estimates.

Participant	Nasion-inion		Tragus-tragus	
	Manual (mm)	MATLAB (mm)	Manual (mm)	MATLAB (mm)
P1	390	391	390	394
P2	348	351	376	380
P3	364	361	368	365
P4	370	368	360	363
P5	352	355	355	353
P6	325	332	377	377

Mean (SD)	358 (22)	360 (20)	371 (13)	372 (15)
